# Targeting EpCAM expression via near-infrared fluorescent antibodies enables microscopic delineation of primary and recurrent HNSCC

**DOI:** 10.1186/s12885-026-16172-2

**Published:** 2026-05-25

**Authors:** Bernd Uhl, Dennis Eggert, Bojan Smiljanov, Katharina von Thun und Hohenstein, Christoph Walz, Gisela Kranz, Sabina Schwenk-Zieger, Florian Haring, Joshua Luft, Jiahang Song, Vincent Holtmann, Philipp Baumeister, Christoph A. Reichel, Olivier Gires, Martin Canis, Christian Betz

**Affiliations:** 1 Department of Otorhinolaryngology, TUM University Hospital, Ismaninger Str. 22, Munich, 81675 Germany; 2 Department of Otolaryngology, University Hospital, Ludwig-Maximilians-Universität München (LMU), Munich, Germany; 3 Walter Brendel Centre of Experimental Medicine, University Hospital, LMU Munich, Munich, Germany; 4 German Cancer Consortium (DKTK), partner site Munich and German Cancer Research Center (DKFZ), Heidelberg, Germany; 5 Department of Otolaryngology – Head and Neck Surgery, University Medical Center Hamburg-Eppendorf, Hamburg, Germany; 6 Institute of Pathology, LMU Munich, Munich, Germany; 7 Comprehensive Cancer Center (CCC) Munich Ludwig-Maximilians- University (LMU), University Hospital, LMU, Munich, Germany; 8 Bavarian Cancer Research Center (BZKF), Munich, Germany

**Keywords:** HNSCC, EpCAM, Image-guided surgery, Fluorescence-guided surgery, Near-infrared, IRDye800

## Abstract

**Background:**

In surgical treatment of head and neck squamous cell carcinoma (HNSCC), resection with adequate tumor-free margins is a fundamental challenge. *Epithelial cell adhesion molecule* (EpCAM/CD326) is substantially overexpressed in HNSCC representing a potential target for fluorescence-guided delineation of HNSCC.

**Materials and methods:**

EpCAM expression was assessed in vitro on squamous cell carcinomas of the upper aerodigestive tract (SCC-UADT) and fibroblasts employing immunostaining with anti-EpCAM antibodies VU1D9 and MT201/adecatumumab (clinically validated). Whole tumor analyses of EpCAM expression were conducted in patient samples with primary and recurrent HNSCC. Confocal microscopy was used for EpCAM expression analyses upon immunostaining in cultured patient HNSCC resection samples using IRDye800CW-labeled MT201.

**Results:**

Immunostaining with VU1D9 and MT201 revealed a high, consistent, and specific expression of EpCAM on SCC-UADT in vitro. Human whole tumor analyses showed high and consistent EpCAM expression (primary: 9/9 > = 80%; recurrent: 6/9 > = 80%) and significantly elevated near-infrared fluorescence intensities for IRDye800CW-VU1D9 and IRDye800CW-MT201 in HNSCC as compared to non-malignant tissue (2–7 fold vs. mucosa; 6–20 fold vs. HNSCC-associated stroma). Translational postoperative immunostaining of live cultured patient HNSCC samples using the clinically validated IRDye800CW-MT201/adecatumumab enabled microscopical differentiation of HNSCC from adjacent non-malignant tissue (fluorescence intensity ratios HNSCC vs. mucosa: 7,66 ± 1,26; and vs. HNSCC-associated stroma: 50,97 ± 8,24)).

**Conclusions:**

IRDye800CW-labeled anti-EpCAM antibodies allow experimental microscopic delineation of HNSCC from non-malignant tissue. As a future outlook, these probes might improve tumor resection in near-infrared fluorescence-guided surgery of primary and recurrent HNSCC.

**Supplementary Information:**

The online version contains supplementary material available at 10.1186/s12885-026-16172-2.

## Introduction/Background

Head and neck cancer is the seventh most common cancer entity worldwide, which are mainly represented by squamous cell carcinoma (HNSCC) [1]. Despite state-of-the-art treatment including surgery, radiotherapy, and/or systemic therapy, survival of patients with advanced disease states is still significantly impaired. A major challenge in surgical therapy of HNSCC is to achieve complete resection of the tumor and marginal invasion zones (resection with wide tumor-free margins), which is associated with considerably decreased cancer recurrence as well as lower patient morbidity and mortality. This applies, in particular, to patients with HNSCC who do not qualify for adjuvant radio(chemo)therapy [2]. While patients benefit the most from tumor-negative margins upon primary resection, they still substantially profit from re-resection in case of initial tumor-positive margins, if tumor-free margins are finally achieved [3]. At present, surgeons make use solely of visual inspection, palpation, as well as their clinical experience together with additional information such as radiological images to completely resect HNSCC tissue with wide margins, i.e. a predominantly recommended 5 mm margin. This is particularly challenging as surgical therapy in the upper aerodigestive tract has to preserve various functionally relevant tissue structures (e.g., for swallowing or talking) to the best of their abilities [4]. Fluorescence-guided surgery represents a promising technique to facilitate intraoperative identification of HNSCC margins, providing the potential to increase rates of tumor-free margin resection as well as functional postoperative outcome [5–8]. Given the promising results of research on neoadjuvant immunotherapy in HNSCC, the demand for reliable techniques to delineate actual tumor borders upon partial immunotherapy response in HNSCC surgery might increase substantially [9]. To date, multiple fluorescent imaging agents have been investigated preclinically as well as in clinical studies (e.g.., indocyanine green (ICG) [10], 5-aminolevulinic acid (5-ALA) [11], epidermal growth factor receptor (EGFR: cetuximab-800CW [12]; panitumumab-IRDye800CW [6]; ABY-029 [13]), vascular endothelial growth factor A (VEGF-A: bevacizumab-IRDye800CW [14]), CD44v6 (IWA-IRDye800CW) [15]), gamma-glutamyltranspeptidase (GGT: gGlu-HMRG [16]), or tumor acidosis (ONM-100 [17])). Translation of fluorescent imaging agents for HNSCC surgery into clinical routine, however, has not been accomplished yet, emphasizing the necessity to identify and validate novel targets [5–8].

The cell surface adhesion and signaling molecule *epithelial cell adhesion molecule* (EpCAM/CD326) is a multi-functional transmembrane protein, essentially regulating cell adhesion, proliferation, migration, stemness, and epithelial-to-mesenchymal transition in various cancer entities [18, 19]. EpCAM is intensively and consistently overexpressed in multiple epithelial cancers including HNSCC, thus representing a potential target for novel fluorescence-guided surgical strategies [18, 20]. In a large cohort of 94 patients, immunohistochemical analyses revealed that roughly 90% of HNSCC exhibit high EpCAM expression [21]. Amongst specific antibodies against EpCAM, the monoclonal mouse anti-human IgG1 antibody VU1D9 shows a high affinity for this molecule and is well-established in the scientific literature [18, 21, 22]. In contrast, the monoclonal human anti-human IgG1 antibody MT201 (adecatumumab), exhibiting an intermediate binding affinity for EpCAM, has been developed by the company Micromet Inc. to generate a therapeutic anti-EpCAM antibody with a favorable toxicity profile, which has been successfully utilized in clinical studies [18, 23]. Regarding fluorescence imaging in surgery, near-infrared fluorescent dyes have been demonstrated to be advantageous because of their superior imaging properties in tissue such as deeper light penetration, reduced light scattering, and diminished background fluorescence. Specifically, the near-infrared dye IRDye800CW has been employed in multiple preclinical- and clinical studies, showing an excellent side effect profile and high-quality fluorescence properties [14, 24, 25].

Consequently, we hypothesized that IRDye800CW-labeled anti-EpCAM antibodies enable fluorescent-guided microscopic differentiation of HNSCC from HNSCC-adjacent non-cancerous mucosa and stroma as a proof-of-concept approach for potential future employment in fluorescence-guided HNSCC surgery.

## Materials and methods

### Ethics statement

The use of human tissue specimen for scientific purposes was approved by the local ethics committee of the Ludwig-Maximilians-Universität (LMU) in Munich (ref. no. 18–446) and the Ethics commission Hamburg (ref. no. WF-049/09). All study participants provided written-informed consent to a clinical study agreeing to share pseudonymized data and samples with research partners located within and outside of the EU. The study has been conducted in accordance with the Declaration of Helsinki and in keeping with the rules of good clinical practice and according to the German laws and ethical standards.

### Anti-EpCAM antibodies

The anti-EpCAM antibodies VU1D9 (monoclonal, high-affinity anti-human mouse IgG1 antibody; # MA1-10195; Thermo Fisher Scientific, Waltham, Massachusetts, USA) and MT201 (adecatumumab; recombinant, monoclonal, moderate-affinity anti-human human IgG1 antibody; courtesy of Micromet Inc., Munich, Germany) were employed for immunostaining of EpCAM. Respective isotype control antibodies were used for VU1D9 (#MA1-10406; Thermo Fisher Scientific, Waltham, Massachusetts, USA) and MT201 (#31154; Thermo Fisher Scientific, Waltham, Massachusetts, USA).

### Antibody labeling with the fluorescent dye IRDye800CW

Murine anti-EpCAM antibody VU1D9 (#MA1-10195; Thermo Fisher Scientific, Waltham, Massachusetts, USA) and respective isotype control antibody (#MA1-10406; Thermo Fisher Scientific, Waltham, Massachusetts, USA) were labeled with fluorescent dye IRDye800CW (Reactive Dye #829–08881; 0.1 mg; Columns and Buffer for Labeling Kits #827–08882; LI-COR Biosciences; Lincoln, Nebraska, USA) according to the manufacturer’s instructions. Using Zeba™ Spin Desalting Columns (#89882; 0.5 ml; Thermo Fisher Scientific, Waltham, Massachusetts, USA), 100 µg of each antibody (1 mg/ml; pH 8.5) were labeled with 0.75 µl dye (1 mg/0,25 ml H20) for 2 h at 20 °C.

Similarly, human, anti-EpCAM antibody MT201 (adecatumumab, Micromet Inc.) and isotype control antibody (#31154; Thermo Fisher Scientific, Waltham, Massachusetts, USA) were labeled with IRDye800CW (LI-COR Biosciences; Lincoln, Nebraska, USA) according to the manufacturer’s instructions. Using Zeba™ Spin Desalting Columns (#89891; 5 ml; Thermo Fisher Scientific, Waltham, Massachusetts, USA), 1000 µg of each antibody (1 mg/ml; pH 8.5) were labeled with 7.5 µl dye (1 mg/0,25 ml H_2_0) for 2 h at 20 °C.

### Cell lines

A human hypopharyngeal squamous cell carcinoma cell line (HSCC; name: FaDu) and a human esophageal squamous cell carcinoma cell line (ESCC; name: KYSE-30) were purchased from DSMZ (Braunschweig, Germany). Human HNSCC-associated fibroblast cell line cancer-associated fibroblast 4 (CAF-4) was established and characterized as described elsewhere in detail [26]. Human foreskin fibroblast cell line HFF-1 was purchased from ATCC (Manassas, USA; SCRC-1041). Cells were cultured in: (i) FaDu: DMEM (Thermo Fisher Scientific, Waltham, Massachusetts, USA) supplemented with 10% fetal bovine serum (FBS) and 1% PenStrep (ThermoFisher Scientific, Waltham, Massachusetts, USA); (ii) KYSE-30: RPMI (Thermo Fisher Scientific, Waltham, Massachusetts, USA) supplemented with 10% FBS and 1% PenStrep (ThermoFisher Scientific, Waltham, Massachusetts, USA); or (iii) CAF-4 & HFF-1: FGM (PromoCell GmbH, Heidelberg Germany) supplemented with fetal calf serum (FCS) 0,02 ml/ml, basic fibroblast growth factor (BFGF) 1 ng/ml, and insulin 5 µg/ml.

### Immunostaining and flow cytometry analyses of cell lines of mucosal squamous cell carcinoma of the upper aerodigestive tract (SCC-UADT) and fibroblasts

In a first step, two mucosal squamous cell carcinoma cell lines of the upper aerodigestive tract (SCC-UADT) – FaDu (HNSCC) and KYSE-30 (ESCC) –, primary HNSCC-associated fibroblasts CAF-4, and normal foreskin fibroblasts HFF-1 were cultivated, harvested, and immunostained with anti-EpCAM antibodies VU1D9 and MT201 or the respective isotype control antibodies for 30 min on ice. After washing, a secondary staining with an AlexaFluor488-labeled anti-human or anti-mouse antibody was performed for 30 min on ice. Afterwards, cells were fixed using lysing/fixation solution (BD Biosciences) for 10 min at room temperature. After washing, immunostained cells were examined on a flow cytometer (Gallios flow cytometer; Beckman Coulter Inc, Brea, California, USA). Approximately 10,000 gated events were collected in each analysis. Isotype-matched controls were used. Isotype-correction of the mean fluorescence intensity values of the anti-EpCAM antibody immunostaining was achieved by subtraction of the respective isotype control mean fluorescence intensities. Data analysis was performed using FlowJo software (Treestar, Ashland, Oregon, USA).

### Cryosections and immunofluorescence staining of bicellular core-shell-spheroids from SCC-UADT and SCC-associated fibroblast cell lines

To test the ability of the anti-EpCAM-antibodies to differentiate SCC-UADT and SCC-associated stroma in a more complex in vitro model, two types of bicellular core-shell-spheroids were generated: (i) one spheroid type with CAF-4 cells as core and HNSCC FaDu cells as shell and (ii) another spheroid type with CAF-4 cells as core and esophageal ESCC KYSE-30 cells as shell. These spheroids were cultured on 1% agarose-coated low attachment 96-well plates (100 µl/well). In a first step, CAF-4 (1 × 10^4^ cells; passage 2–3) were plated separately in 100 µl DMEM (10% FCS) per well to create monocellular fibroblast spheroids. After 3 days, core-shell-spheroids were produced by co-culturing SCC cells (2.5 × 10^3^; FaDu or KYSE-30) with CAF-4 spheroids for further 7 days while renewing the medium every 48 h. After harvesting, core-shell-spheroids were embedded in Tissue-Tek^®^ O.C.T.™ (Sakura Finetek, Torrance, CA), shock-frozen in liquid nitrogen, and stored at − 20 °C. Cryosections (thickness: 5 μm) were generated on a cryostat (Leica CM 1900; Deerfield, Illinois, USA) and fixed in acetone. The tissue sections were then immunostained with a combination of (i) S100A4 combined with a secondary AlexaFluor488-labeled goat anti-rabbit antibody (ab15077; abcam, Cambridge, UK), and (ii) either the IRDye800CW-labeled anti-EpCAM antibody VU1D9 (murine) or MT201 (human) or the respective isotype control antibodies for 16 h at 4°C and mounted in ProLong™ Diamond Antifade Mountant with (iii) DAPI (#P36962; Thermo Fisher Scientific, Waltham, Massachusetts, USA).

### Cryosections of tissue samples of patients with primary and recurrent HNSCC

Patient sample pairs of primary or recurrent HNSCC and non-malignant mucosa – as histopathologically diagnosed by the Institute of Pathology of the LMU Munich – were embedded in OCT (TissueTec) and subsequently frozen. Samples were randomly harvested without prescreening from patients undergoing surgery for HNSCC and consented to the study in the department of otorhinolaryngology of the university clinic of LMU Munich. Next, cryosections were generated (thickness: 5 µm) on a cryostat (Leica; Deerfield, Illinois, USA) and fixed in acetone. In a first step, semi-quantitative analyses of the EpCAM expression were performed by an experienced pathologist of the Institute of Pathology of the LMU Munich in the entire tumor tissue visible in these cryosections after hematoxylin staining of nuclei and anti-EpCAM (VU1D9) staining. In additional cryosections of the same tissue samples, two-color immunostaining was performed for epifluorescence examinations: The tissue sections were incubated with either IRDye800CW-labeled anti-EpCAM antibodies VU1D9 or MT201 or the respective isotype control antibodies for 16 h at 4 °C and subsequently mounted in ProLong™ Diamond Antifade Mountant, which includes DAPI (#P36962; Thermo Fisher Scientific, Waltham, Massachusetts, USA).

### Multi-channel epifluorescence microscopy on cryosections of bicellular SCC-UADT/CAF-4-spheroids and tissue samples of patients with primary and recurrent HNSCC

Multi-channel epifluorescence microscopy was conducted with an Axio Scope.A1 microscope (Zeiss MicroImaging GmbH, Goettingen, Germany), equipped with two different LED light sources for fluorescence epi-illumination and a combiner for parallel usage: (i) a Colibiri.2 LED light source (Zeiss MicroImaging GmbH) and (ii) a near-infrared (NIR) LED light source (pE-100, CoolLED, Andover, England). For excitation and discrimination of the fluorescent emissions, LED light was used either from the first source (365 nm, 470 nm, 555 nm, or 625 nm) in combination with a QUAD filter set (QUAD DAPI (4′,6-diamidino-2-phenylindole)/FITC/Cy3/Cy5 sbx HC Filterset; AHF Analysentechnik AG, Tübingen, Germany) or from the second source (770 nm) in combination with a IRDye800CW filter set (Licor IR Dye 800 HC Filterset (Langpass); AHF Analysentechnik AG, Tübingen, Germany). Microscopy images were obtained with an AxioCam Hsm digital camera using 10 × (0.7 NA) or 40 × (1.0 NA) water immersion lenses (Zeiss MicroImaging Gmbh). The images were processed with AxioVision software (Zeiss MicroImaging GmbH). The software Fiji (distribution of the software ImageJ) was used for offline analysis of images to determine mean fluorescence intensities of the IRDye800CW-coupled anti-EpCAM-antibodies or the respective isotype control antibodies in the identified regions of interest.

### Tissue culture of intraoperatively harvested tissue samples of HNSCC patients

Live, fresh tissue pairs (HNSCC and non-malignant mucosa) of 14 patients were collected during surgery under general anesthesia. All patients with histopathological confirmed primary or recurrent HNSCC undergoing surgical therapy in the department of otorhinolaryngology of the University Medical Center Hamburg-Eppendorf, who consented to the study, were included. Exclusion criteria were: (i) patients under 18 years of age, (ii) patients incapable of giving consent, (iii) pregnant and breastfeeding women, and (iv) persons of childbearing potential without contraception. Patients were *not* pre-screened with regard to HNSCC EpCAM expression or further specific tumor characteristics to evaluate the general employability of IRDye800CW-MT201 in HNSCC.

Tissue samples were stored in tissue culture medium at 37 °C to maintain tissue samples alive. The medium was based on Dulbecco’s modified Eagle medium (DMEM, Gibco, Darmstadt, Germany) supplemented with 10% heat inactivated (65 °C; 30 min) FBS (Biochrome, Berlin, Germany), 1% Penicillin/Streptomycin and 1% Amphotericin B (both Gibco). Cultivation of tumor slices largely followed the method described by Berger et al. for HNSCC [27]. Slice cultures were incubated at the air–liquid-interface at 37 °C with 10% CO_2_ and 100% humidification overnight for recovery and re-oxygenation. Subsequently, tissue samples were first cut with a scalpel and then cut into 400 μm slices using a MacIllvine tissue chopper (The Mickle Laboratory Engineering Co. Ltd, Gomshall, UK) or, in case the texture did not allow machine cutting, using a scalpel. Tissue slices were then placed on Millicell^®^ cell culture inserts (0.4 μm, 30 mm diameter, Merck KGaA, Darmstadt, Germany) in 6-well dishes containing the tissue culture medium. The tissue slices were cultivated for up to two days, the medium was exchanged every 24 hours.

### Postoperative fluorescence staining of intraoperatively harvested live tissue samples of HNSCC patients

Antibody staining was performed by diluting antibody solutions (IRDye800CW-labeled MT201 antibody or AlexaFluor488-labeled MT201 antibody or the respective isotype control antibodies) directly into culture medium at a dilution of 1:100. After 4 h, samples were washed once with pre-warmed phosphate buffered saline (PBS, Gibco). Nuclei were stained with DAPI (Thermo Fisher Scientific, Waltham, Massachusetts, USA) by adding the DAPI stain solution into the culture medium (final concentration 300 nM). After 5 min, samples were washed once with pre-warmed PBS. The membranes of the cells were stained with CellMask™ Green Plasma Membrane Stain (Thermo Fisher Scientific) by adding the stain solution into the culture medium according to the manufacturer’s protocol. After 5 min, samples were washed once with pre-warmed PBS.

### Confocal microscopy analyses of intraoperatively harvested tissue samples of HNSCC patients after ex vivo immunostaining

Confocal microscopy was performed using a Nikon A1 confocal microscope (Nikon GmbH, Düsseldorf, Germany) equipped with four lasers (405 nm, 488 nm, 561 nm, and 640 nm) and an incubation chamber. The chamber was heated to 37 °C with 90% humidity. Either a 10x air objective (Plan Apo Lambda 10x, NA 0.45 – Nikon GmbH) or a 20x air objective (Plan Apo Lambda 20x, NA 0.80 – Nikon GmbH) were used for imaging. For discrimination of the fluorescent emissions, a suited quad bandpass filter set (LED-DA/FI/TR/Cy5/Cy7a – Semrock, Inc., Rochester, NY, USA) was used. The NIS Elements AR software package (v. 5.21; Laboratory imaging s.r.o., Prague, Czech Republic) and the Imaris software package (v. 7.6.1; Bitplane, Belfast, United Kingdom) were used for measurements and the visualization of the images.

### Analysis of TCGA RNA sequencing data for association of EpCAM RNA levels with clinicopathological characteristics of HNSCC

RNA sequencing raw count data and corresponding clinicopathological information were obtained from The Cancer Genome Atlas (TCGA) database. Raw counts were normalized to counts per million (CPM) to account for differences in library size across samples, and CPM values were log2-transformed for downstream analyses. EpCAM RNA levels were extracted from the processed dataset and matched with available clinical parameters HPV status, tumor site, histological grade, clinical stage, T stage, and lymph node status. Samples with incomplete clinical information were excluded from the corresponding subgroup analyses. Differences in EpCAM RNA levels between two groups were evaluated using the Wilcoxon rank-sum test. For comparisons involving more than two groups, pairwise Wilcoxon rank-sum tests were performed. *P* values from multiple comparisons were adjusted using the Benjamini-Hochberg method to control the false discovery rate. All statistical tests were two-sided, and a *p* value < 0.05 was considered statistically significant.

### Statistics

Data analysis was performed with the statistical software SigmaPlot for Windows (Jandel Scientific, Erkrath, Germany). After confirming normality and equal variance of data using the Shapiro-Wilk and Brown-Forsythe tests, One-way ANOVA test followed by the Dunnett test (> 2 groups) or the *t* test (two groups) were used for the estimation of stochastic probability in intergroup comparisons. If normality and/or equal variance testing failed, the Kruskal-Wallis One-way ANOVA of Ranks test followed by the Dunnett test (> 2 groups) or the Mann-Whitney rank sum test (two groups) were used. *P* values < 0.05 were considered significant.

## Results

### EpCAM expression in cell lines of SCC-UADT and (SCC-associated) fibroblasts

**Fig. 1:**
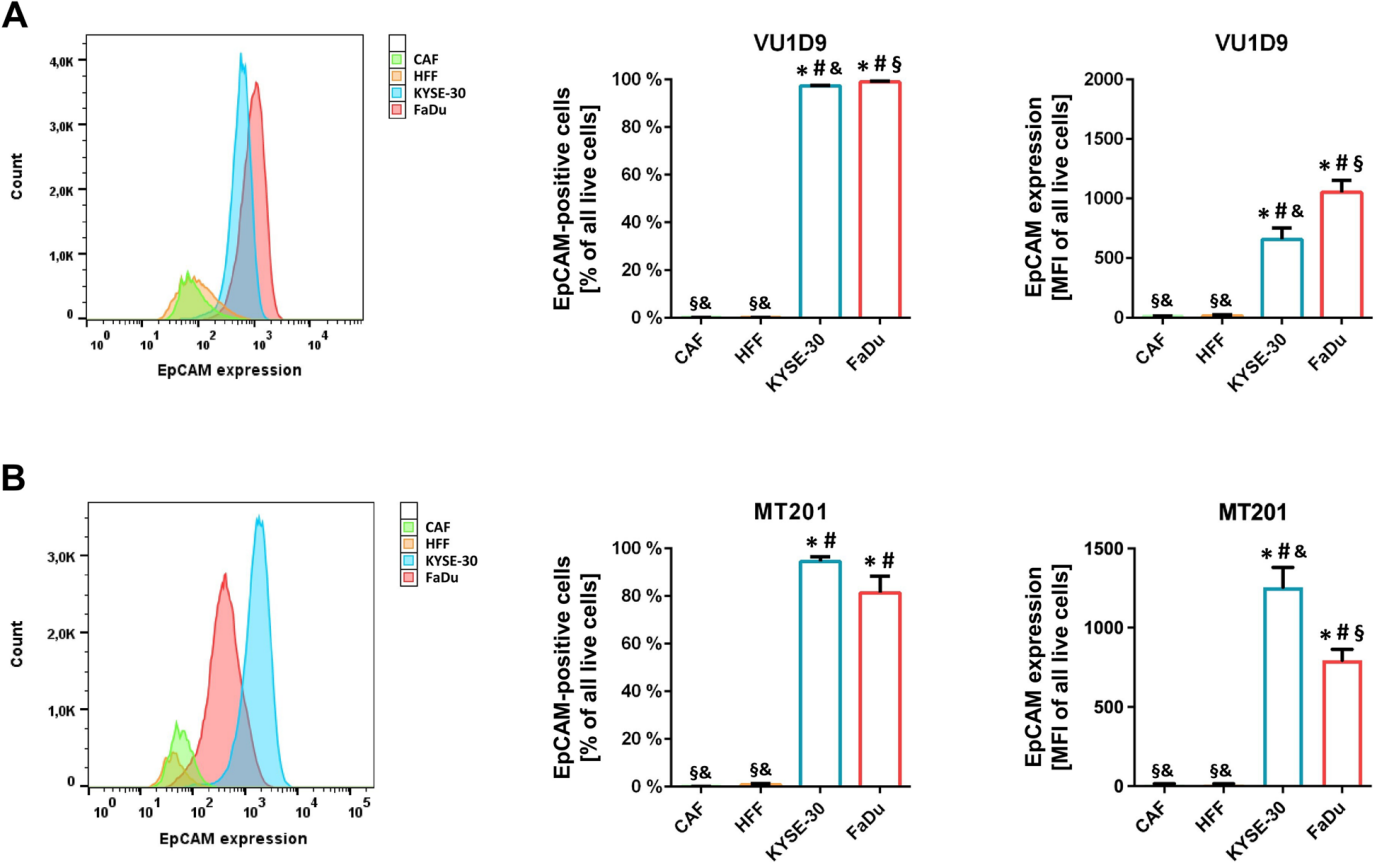
**Expression of EpCAM on cell lines of SCC-UADT, HNSCC-associated fibroblasts, and normal fibroblasts.** **A** Representative histogram and quantitative data on the percentage of EpCAM-positive cells as well as on the expression level of EpCAM of the SCC-UADT cell lines KYSE-30 (ESCC) and FaDu (HNSCC), the HNSCC-associated fibroblast cell line CAF-4, and the normal fibroblast cell line HFF-1 as assessed by flow cytometry upon immunostaining with the AlexaFluor488-labeled anti-EpCAM antibody VU1D9 (high EpCAM-affinity) (mean ± SEM for *n* = 4; * *p* < .05 vs. CAF; # *p* < .05 vs. HFF; § *p* < .05 vs. KYSE-30; & *p* < .05 vs. FaDu). **B** Representative histogram and quantitative data on the percentage of EpCAM-positive cells as well as on the expression level of EpCAM of the SCC-UADT cell lines KYSE-30 and FaDu, the HNSCC-associated fibroblast cell line CAF-4, and the normal fibroblast cell line HFF-1 as assessed by flow cytometry upon immunostaining with the AlexaFluor488-labeled anti-EpCAM antibody MT201 (intermediate EpCAM-affinity; clinically validated) (mean ± SEM for *n* = 4; * *p* < .05 vs. CAF; # *p* < .05 vs. HFF; § *p* < .05 vs. KYSE-30; & *p* < .05 vs. FaDu)

In a first step, we analyzed EpCAM expression in the two well-established SCC-UADT cell lines FaDu (HNSCC) and KYSE-30 (ESCC) as well as HNSCC-derived CAF-4 [26] and normal foreskin fibroblasts HFF-1. Our data revealed that > 95% of the KYSE-30 and FaDu cells are EpCAM-positive as detected by VU1D9 antibody (high EpCAM-affinity; Fig. 1A), while > 90% of the KYSE-30 cells and > 80% of the FaDu cells are EpCAM-positive as detected by MT201 antibody (moderate EpCAM-affinity; Fig. 1B). EpCAM-positive FaDu cells demonstrated an isotype control-corrected relative fluorescence intensity of 1053.33 ± 107.50 (VU1D9) or of 794.05 ± 139.12 (MT201), while EpCAM-positive KYSE-30 cells showed an isotype control-corrected relative fluorescence intensity of 658.00 ± 190.34 (VU1D9) or of 1256.13 ± 248.17 (MT201). In contrast, CAF-4 and normal HFF-1 fibroblasts did not exhibit relevant EpCAM expression as evaluated by staining with both anti-EpCAM antibodies (CAF-4: 11.29 ± 13.06 (VU1D9); 9.5 ± 11.04 (MT201); HFF-1: 17.65 ± 20.38 (VU1D9); 10.08 ± 12.08 (MT201). Hence, EpCAM is validly detectable *via* antibody immunofluorescence staining and is highly and consistently expressed on SCC-UADT cells, but not on (HNSCC-associated) fibroblasts.

### EpCAM expression patterns in bicellular core-shell-spheroids of SCC-UADT and CAF-4 cells

**Fig. 2:**
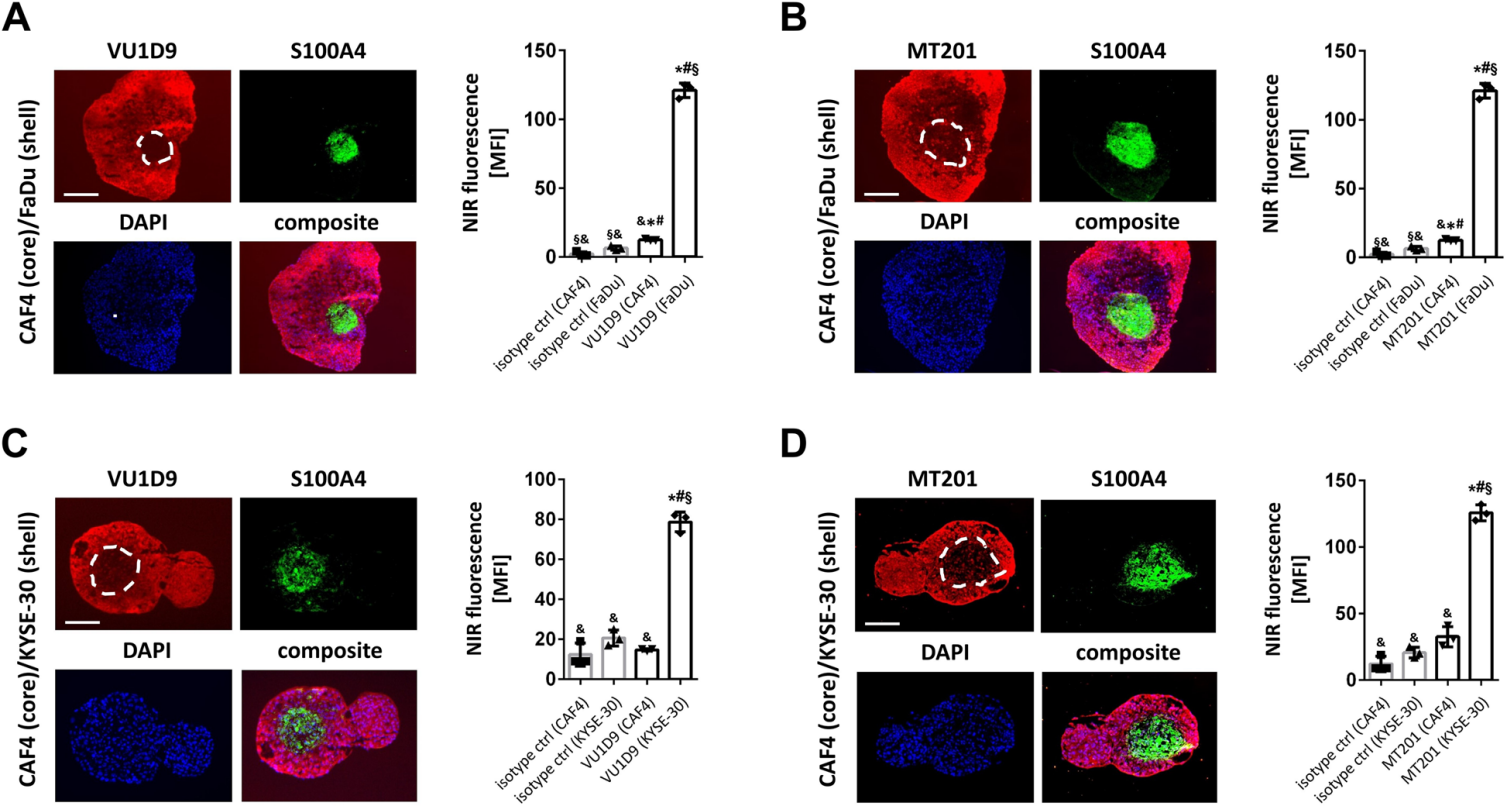
** A**,** B** Representative multi-color epifluorescence microscopy images of immunohistochemically stained cryo-sections of bicellular core-shell-spheroids of HNSCC (FaDu; shell) and HNSCC-associated fibroblasts (CAF-4; core) illustrating the expression of EpCAM (red), S100A4/fibroblasts (green), DAPI/DNA (blue; scale bar: 200 μm; white dashed line: border FaDu/CAF-4) as well as quantitative data on the mean fluorescence intensity of the immunostaining with the IRDye800CW-labeled anti-EpCAM antibody VU1D9 (**A**) or MT201 (**B**) or the respective control antibodies in the HNSCC shell as well as the HNSCC-associated fibroblast core (CAF-4) (mean ± SEM for *n* = 3; * *p* < .05 vs. isotype ctrl (CAF4); # *p* < .05 vs. isotype ctrl (FaDu); § *p* < .05 vs. VU1D9 (CAF4) or MT201 (CAF4) ; & *p* < .05 VU1D9 (FaDu) or MT201 (FaDu)). **C**,** D** Representative multi-color epifluorescence microscopy images of immunohistochemically stained cryo-sections of bicellular core-shell-spheroids of ESCC (KYSE-30; shell) and HNSCC-associated fibroblasts (CAF-4; core) illustrating the expression of EpCAM (red), S100A4/fibroblasts (green), DAPI/DNA (blue; scale bar: 200 μm; white dashed line: border KYSE-30/CAF-4) as well as quantitative data on the mean fluorescence intensity of the immunostaining with the IRDye800CW-labeled anti-EpCAM antibody VU1D9 (**C**) or MT201 (**D**) or the respective control antibodies in the ESCC shell as well as the HNSCC-associated fibroblast core (CAF-4) (mean ± SEM for *n* = 3; * *p* < .05 vs. isotype ctrl (CAF4); # *p* < .05 vs. isotype ctrl (KYSE-30); § *p* < .05 vs. VU1D9 (CAF4) or MT201 (CAF4) ; & *p* < .05 VU1D9 (KYSE-30) or MT201 (KYSE-30))

Employing the in vitro model of bicellular core-shell-spheroids, we found a manifold increased mean fluorescence intensity of the S100A4-negative SCC region as compared to the S100A4-positive CAF-4 region for both SCC-UADT cell lines and both IRDye800CW-coupled anti-EpCAM antibodies (VU1D9: Fig. 2A/2C and MT201: Fig. 2B/2D). Notably, the CAF-4 region itself exhibited a slightly increased mean EpCAM fluorescence intensity as compared to background signal, which was largely based on single HNSCC cells infiltrating the fibroblast region (Supp. Fig. 3). Thus, immunostaining of HNSCC *via* anti-EpCAM-antibodies allows to differentiate SCC-UADT tissue from SCC-associated connective tissue in multicellular models of UADT/stroma.

**Fig. 3:**
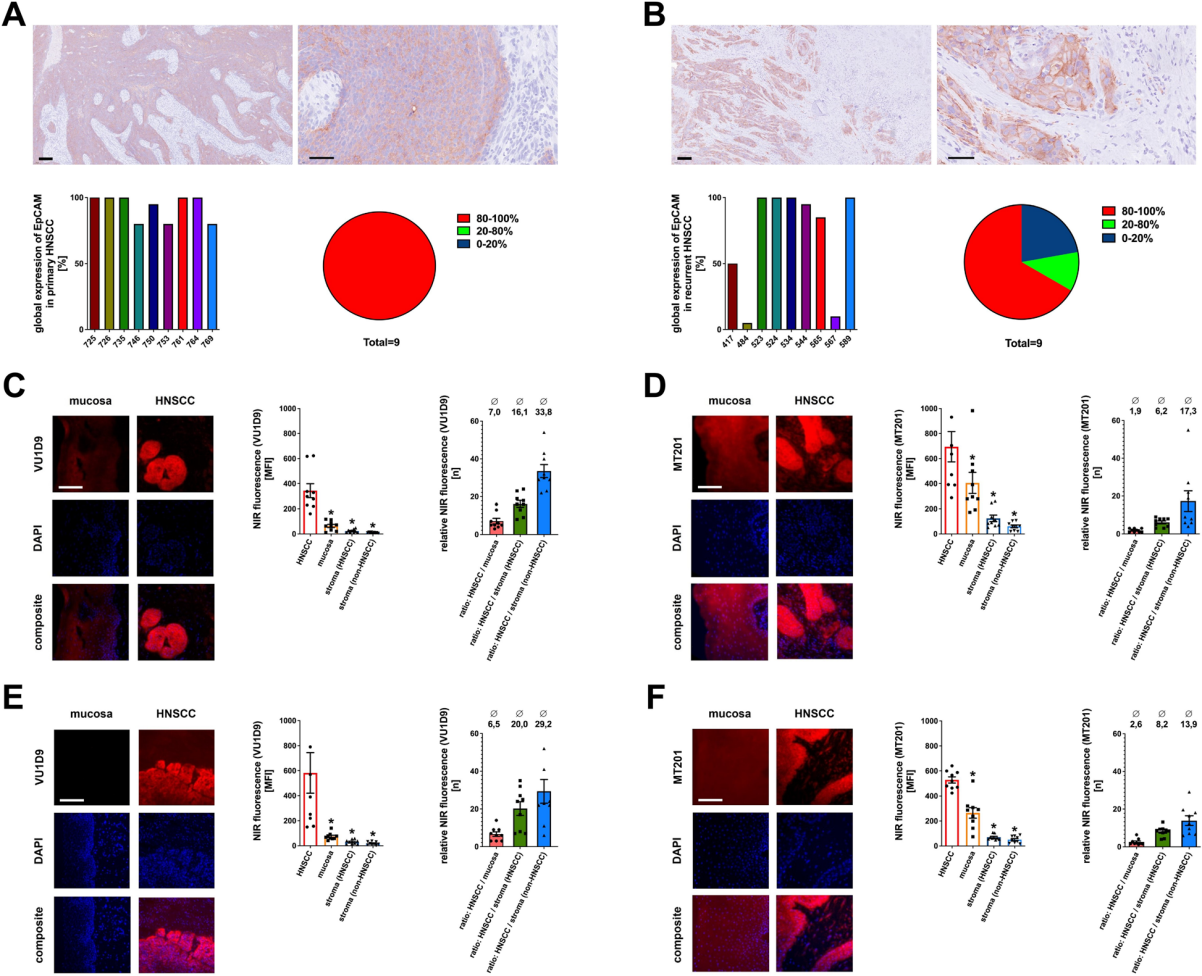
**EpCAM expression in HNSCC, non-malignant HNSCC-associated and non-HNSCC-associated stroma, as well as non-malignant mucosa in cryosections of patient tissue samples with primary and recurrent HNSCC. A**,** B** Representative light microscopy images (overview, scale bar: 200 μm; right: details, scale bar: 60 μm) of immunohistochemically stained HNSCC tissue (hematoxylin staining of nuclei; anti-EpCAM-antibody VU1D9: red) as well as quantitative data on the percentage of EpCAM-positive HNSCC tissue of all the HNSCC tissue visible in the patient tissue samples with primary (**A**) and recurrent (**B**) HNSCC (*n* = 9 per group) upon immunohistochemical EpCAM**/**hematoxylin staining. Quantitative data is illustrated as absolute expression percentages (i) of the single patient samples as bar chart and (ii) grouped as pie chart (expression percentage 0–19%: blue; 20–79%: green; 80–100%: red). **C-F** Representative multi-color epifluorescence microscopy images of immunohistochemically stained additional cryosections of the same tissue samples of HNSCC and of non-malignant mucosa illustrating the expression of EpCAM (red) and DAPI/DNA (blue; scale bar: 100 μm) as well as quantitative data on the relative background-corrected mean fluorescence intensity levels of selected regions of interest (HNSCC, non-malignant mucosa, HNSCC-associated stroma, and non-HNSCC-associated stroma (subepithelial)) as assessed by anti-EpCAM antibody immunostaining with the antibody VU1D9 (**C/E**) and MT201 (**D/F**) in tissue samples of patients with primary (**C/D**) and recurrent (**E/F**) HNSCC (mean ± SEM for *n* = 9; * *p* < .05 vs. HNSCC)

### EpCAM expression in human primary and recurrent HNSCC as well as non-malignant mucosa/stroma

Analyses of the entire tumor area in cryosections of patients with HNSCC revealed high and consistent EpCAM expression patterns in all primary HNSCCs (9/9: >= 80%; 0/9: 21–79%; 0/9: <=20%; Fig. 3A) as well as the majority of recurrent HNSCCs (6/9: >= 80%; 1/9: 21–79%; 2/9: <=20%; Fig. 3B). Apart from HNSCC tissue, relevant EpCAM expression was found in salivary gland tissue and low EpCAM expression in non-malignant mucosa, primarily in suprabasal cells (Supp. Fig. 1A/B). Noteworthy, some HNSCCs exhibited central cornification areas with lower EpCAM expression. In fluorescence immunostaining experiments with both IRDye800CW-labeled anti-EpCAM-antibodies in additional, adjacent cryosections of the same tissue samples, we quantified the background-corrected fluorescence intensity of patient-matched HNSCC-tissue, HNSCC-associated stroma, as well as non-malignant mucosa. Our data show that the mean fluorescence intensity in primary (Fig. 3C/3D) and recurrent HNSCC (Fig. 3E/3F) is in average significantly higher than in non-malignant mucosa (MT201: primary: 2 times higher, recurrence: 3 times higher; VU1D9: primary: 7 times higher, recurrence: 7 times higher) and several times higher as in HNSCC-associated stroma (MT201: primary: 6 times higher, recurrence: 8 times higher; VU1D9: primary: 16 times higher, recurrence: 20 times higher). Collectively, the VU1D9-antibody enables to differentiate HNSCC from non-malignant mucosa and HNSCC-associated stroma, while the MT201-antibody also allows to clearly differentiate HNSCC from HNSCC-associated stroma, but only moderately distinguishes between HNSCC and non-malignant mucosa.

### IRDye800CW-MT201 immunostaining of live tissue enables fluorescence-based differentiation of human HNSCC and HNSCC-associated stroma

**Fig. 4:**
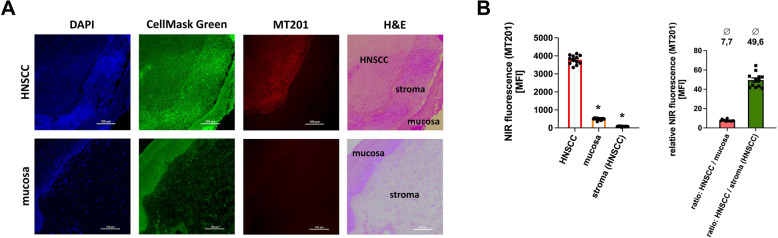
**Postoperative immunostaining of live intraoperatively harvested and cultured patient samples with the IRDye800CW-labeled anti-EpCAM-antibody MT201 (adecatumumab) for fluorescence-based differentiation of HNSCC, non-malignant mucosa, and non-malignant HNSCC-associated stroma. A** Representative multi-color confocal laserscanning microscopy images of live intraoperatively harvested and postoperatively immunostained paired tissue sample of HNSCC and non-malignant mucosa (DAPI/DNA: blue; CellMask Green/cell membranes: green; MT201/EpCAM: red; Corresponding H&E staining: multicolor; scale bar: 100 μm) as well as (**B) **quantitative data on the relative background-corrected mean fluorescence intensity levels of selected regions of interest (HNSCC, non-malignant mucosa, and non-malignant HNSCC-associated stroma) as assessed by immunostaining with the anti-EpCAM antibody MT201 (adecatumumab) in tissue samples of patients with primary and recurrent HNSCC (mean ± SEM for *n* = 12; * *p* < .05 vs. HNSCC)

Confocal laser scanning microscopy of postoperatively stained live tissue samples showed a defined near-infrared fluorescence staining of the HNSCC cells. Non-malignant mucosa and HNSCC-associated stroma both demonstrated a several times lower mean fluorescence intensity as compared to HNSCC tissue (Fig. 4A/4B). To gain further insight in the quality of the construct of the NIR dye IRDye800CW with the anti-EpCAM-antibody MT201, we compared the imaging properties of a standard dye in the visible light spectrum (AlexaFluor488-labeled anti-EpCAM-antibody MT201) to the NIR dye employed in this project (IRDye800CW-labeled anti-EpCAM-antibody MT201). Expectedly, the usable imaging depth was considerably higher for the NIR stain as compared to the stain in the visible light spectrum (Supp. Fig. 2A). Moreover, immunostaining with the NIR dye-labeled antibody resulted in a significantly higher intensity ratio for HNSCC to non-malignant mucosa (7,66 ± 1,26 (IRDye800CW) vs. 2,42 ± 0,52 (AlexaFluor488)) and HNSCC to HNSCC-associated stroma (50,97 ± 8,24 (IRDye800CW) vs. 4,12 ± 0,79 (AlexaFluor488) as compared to the staining in the visible light spectrum (Supp. Fig. 2A).

### Association between EpCAM RNA levels and relevant clinicopathological characteristics

The TCGA dataset analysis revealed relevant EpCAM RNA levels in the majority of HNSCC for all clinicopathological characteristics. However, RNA levels in HNSCC exhibit significant variability. Noteworthy, specific clinicopathological HNSCC characteristics are associated with higher EpCAM RNA levels: EpCAM RNA levels were significantly higher in HPV-positive tumors than in HPV-negative tumors (*p* < .001). Among different tumor sites, EpCAM RNA levels were significantly lower in oral cavity tumors as compared to oropharyngeal and laryngeal tumors (*p* < .001), whereas no significant differences were observed among the other sites. With respect to histological grade, EpCAM RNA levels were significantly higher in G2–G4 tumors compared with G1 (*p* < .05), while no significant differences were detected among G2, G3, and G4. EpCAM RNA levels increased with advancing clinical stage. Stage II–IV tumors showed significantly higher expression than Stage I (*p* < .05), whereas differences among Stages II–IV were not statistically significant. No significant association was observed between EpCAM RNA levels and T stage. However, EpCAM RNA levels were significantly higher in patients with lymph node metastasis compared with those without (*p* < .001).

## Discussion

Fluorescence-guided surgery has the potential to considerably improve complete surgical resection of HNSCC, which is critical to control tumor recurrence and enhance patient survival [5–8, 15]. Here, we systematically evaluated cell surface adhesion and signaling molecule EpCAM as target for ex vivo near-infrared immunofluorescence-guided microscopic delineation of HNSCC against non-cancerous tissue.

In a first set of experiments, we employed two different well-established, specific, and fluorescence-labeled anti-EpCAM antibodies for detection of this molecule in permanent cell lines: High-affinity anti-EpCAM antibody VU1D9 and moderate-affinity anti-EpCAM, but clinically validated antibody MT201 (adecatumumab). Our results reveal a high and consistent as well as in comparison to HNSCC-associated and normal fibroblasts specific expression of EpCAM on two different SCC-UADT cell lines (FaDu: HNSCC; KYSE-30: ESCC), concomitantly confirming antibody-targeting as applicable method for EpCAM visualization. To study the expression patterns of EpCAM in HNSCC and adjacent stroma in a three-dimensional context, we subsequently analyzed cryo-sections of bicellular core-shell spheroids grown from HNSCC or ESCC and HNSCC-associated, primary fibroblasts employing immunostainings with IRDye800CW-labeled VU1D9 and MT201 antibodies. Similar to immunostaining of individual cell types, we found a high and consistent fluorescence signal in the SCC shell region for both antibodies, while only a marginal fluorescence signal was detectable in the fibroblast core region. Noteworthy, we were able to microscopically identify single HNSCC cells invading the tumor fibroblast core region. This finding is of particular interest as it indicates that immunostaining with IRDye800CW-labeled anti-EpCAM antibodies might enable detection of residual tumor cells and tumor buds comprised of single or very few cells. Tumor budding as well as postoperative minimal residual disease in HNSCC are prognostic of poorer survival and a thorough intraoperative detection would thus be beneficial [28, 29]. Previously published data on EpCAM expression in HNSCC correspondingly demonstrate a significant expression on 11 of 12 examined HNSCC cell lines: while the average relative expression levels as compared to controls varied between ~ 1 and 9, a relatively high expression level of around 3 or above has been found for 8 of 12 cell lines as analyzed by western blotting [30]. These data emphasize the potential of IRDye800CW-labeled anti-EpCAM antibodies for fluorescence-guided microscopic delineation of HNSCC versus HNSCC-associated tissue.

Intraoperative applications of the IRDye800CW-labeled anti-EpCAM-antibodies for fluorescence-guided HNSCC surgery, however, require clear delineation of HNSCC from all non-malignant structures of the surrounding tissue in the complex three-dimensional HNSCC environment. To address this challenge, we first performed anti-EpCAM immunostainings in matched pairs of (i) primary or recurrent HNSCC with adjacent stroma and (ii) samples of non-malignant mucosa from the same patients. Semi-quantitative expression analyses substantiated a high and consistent EpCAM expression (> 80%) in all primary HNSCCs and in the majority of recurrent HNSCCs. The observed lower EpCAM expression in recurrent HNSCC might be a consequence of temporal tumor evolution including genetic, functional and phenotypic alterations [5]. Comparable to renowned targets for fluorescence-guided HNSCC surgery such as EGFR, CD44v6, or integrin αVβ6, significant EpCAM expression was also observed in non-cancerous salivary gland tissue (moderate) and in non-malignant mucosa (low) representing a potential limitation for EpCAM-based fluorescence-guided HNSCC delineation [15, 31–35]. Surgeons should be particularly aware of possible false-positive fluorescence signals in salivary glands, which might limit the use of anti-EpCAM probes in HNSCC margin assessment of tumors infiltrating into salivary glands.

In further immunostainings of these human HSNCC samples, we found that the IRDye800CW-labeled, high-affinity anti-EpCAM antibody VU1D9 allows precise differentiation of HNSCC from adjacent stroma and non-malignant mucosa. The clinically applicable, IRDye800CW-labeled, and moderate-affinity anti-EpCAM antibody MT201 similarly enables to distinguish clearly between HNSCC and HNSCC-associated stroma, but only moderately between HNSCC and non-malignant mucosa. Former studies have correspondingly shown a generally high and broad expression of EpCAM in HNSCC as assessed by RNA-Seq (TCGA-HNSCC dataset) and/or immunohistochemical staining [20, 36], while other authors described substantial EpCAM expression only in 60% of HNSCC associated with a higher stromal expression in immunohistochemical staining of tissue slices [15]. EpCAM expression in non-malignant mucosa, analogous to our results, has been shown to be absent or only weak in previous studies [37]. Noteworthy, one group has reported a moderately elevated expression in non-malignant, hyperplastic tumor-adjacent mucosa in the marginal zone as compared to ‘healthy’ mucosa whereas the expression was still lower than in HNSCC tissues [38]. These data collectively indicate that NIR-fluorescent anti-EpCAM immunostaining might generally be suitable for microscopic delineation of HNSCC from non-malignant mucosa and particularly submucosal HNSCC-associated stroma. However, the differentiation level, especially for HNSCC *versus* non-malignant mucosa, varied in dependence of the employed antibody clone. As regards HNSCC with limited EpCAM expression, preoperative tumor mapping with representative tissue samples might help to evaluate the applicability of an anti-EpCAM immunostaining for image-guided surgery and exclude patients with EpCAM-low HNSCC. Another approach to address the expression heterogeneity of cancers for specific targets might be to combine different, complementing targeted fluorescent agents thereby enhancing complete coverage and potentially the fluorescence intensity of an HNSCC staining for image-guided surgery. To examine the potential of target combinations for fluorescence-guided HNSCC surgery as well as to evaluate the effectiveness of EpCAM as single target in comparison with the currently most promising candidates (e.g., EGFR, CD44v6, integrins), further experiments would be advisable ideally in a direct experimental comparison of those targets in one HNSCC sample cohort.

In a translational proof-of-concept approach, postoperative immunostaining in intraoperatively harvested, live cultured patient tissue samples with primary and recurrent HNSCC with the clinically validated IRDye800CW-MT201 revealed that microscopic analysis enables to differentiate HNSCC from non-malignant tissues with a tumor-to-background ratio (TBR) of 5–10 for the superficial mucosal HNSCC margin and of > 40 for the deep stromal HNSCC margin [39]. These experimental TBRs are encouraging considering a formerly hypothesized minimal TBR of 1.5 required for adequate differentiation under ideal circumstances [39] and the given TBRs of other promising preclinically and clinically tested imaging agents in the range of 2–5 [15, 17, 40]. As a future outlook, perioperative ex vivo immunostaining of live tissue samples with IRDye800CW-MT201 might enable surgeons to differentiate HNSCC from non-malignant tissue and thus identify tumor-positive margins in fluorescence-guided surgery. Such intraoperative applications, however, would require comparative analyses of the EpCAM-based NIR immunostaining approach with classical histopathological analysis for HNSCC delineation. Moreover, the immunostaining protocol utilized in this study (time consumption of ~ 4 h) would need to be substantially optimized to have shorter staining periods. In current clinical studies for fluorescence-guided surgery, staining times were described to be, e.g., below 0.5 h (γ-glutamyltranspeptidase-activated targeting agent) or below 3 h (5-aminolevulinic acid) [16, 41]. Beyond the employed ex vivo immunostaining, our antibody-dye construct IRDye800CW-MT201 might also be applicable for in vivo fluorescence-guided surgery upon systemic infusion, as both, the anti-EpCAM antibody MT201 and the fluorescent dye IRDye800CW, have already been tested in clinical studies revealing a decent side effect profile [14, 18, 23–25].

In a last set of experiments, we sought to identify whether specific HNSCC subsets as defined by major clinicopathological parameters might be particularly suitable or, in fact, particularly unsuitable for EpCAM-based HNSCC delineation. EpCAM RNA levels as indicator for EpCAM expression were analyzed in the large HNSCC dataset of "The Cancer Genome Atlas" (TCGA) demonstrating relevant EpCAM RNA levels in the vast majority of HNSCC, althought the absolute RNA levels exhibited significant variability. While all subgroups appear to be generally suitable for EpCAM-based identification of HNSCC, certain subgroups might be particularly well suited due to their collectively higher and less variable RNA expression levels: HPV-positive HNSCC, less differentiated HNSCC (as defined by higher histopathological grading), HNSCC in higher UICC stages, HNSCC with lymph node metastasis, as well as HNSCC of Oropharynx and Larynx. Noteably, this RNA-based bulk data analysis requires experimental confirmation on the protein level such as by immunostaining approaches.

As subsequent steps toward clinical translation, the authors suggest evaluating the anti-EpCAM antibody-dye constructs IRDye800CW-MT201 and IRDye800CW-VU1D9 for intraoperative ex vivo margin assessment using NIR microscopy on whole HNSCC resection samples employing immunostaining with practicable incubation times of, e.g., 30, and/or 60 min in direct comparison to classical histopathological analysis. In case of successful validation in the ex vivo setting, the antibody-dye constructs should furthermore be tested for intraoperative in vivo margin assessment, ideally in surgical systems with integrated NIR-capable camera systems such as in transoral laser microsurgery (TLM) or transoral robotic surgery (TORS).

## Conclusions

In conclusion, we demonstrated in in vitro experiments and human tissue samples that immunostaining with NIR-fluorescent IRDye800CW-labeled anti-EpCAM antibodies VU1D9 and MT201 (adecatumumab) allows clear microscopic differentiation of HNSCC tissue from adjacent non-malignant HNSCC stroma and mucosa. The relevance EpCAM expression in non-HNSCC salivary gland tissue (moderate) and mucosa (low) as potential limitation warrants further detailed investigation. As a future outlook, particularly the antibody-dye construct IRDye800CW-MT201 might be a promising candidate for follow-up studies assessing its intraoperative applicability for ex vivo or in vivo fluorescence-guided surgery to potentially enhance margin assessment and concomitantly complete cancer resection in primary and recurrent HNSCC.

## Supplementary Information

Supplementary Material 1.

## Data Availability

The datasets supporting the conclusions of this article are included within the article and its additional file.

## Abbreviations

5-ALA: 5-aminolevulinic acid
BFGF: Basic fibroblast growth factor
CAF-4: Cancer-associated fibroblasts 4 (HNSCC-associated)
CO2: Carbon dioxide
DNA: Deoxyribonucleic acid
EGFR: Epidermal growth factor receptor
EpCAM: Epithelial cell adhesion molecule
ESCC: Esophageal squamous cell carcinoma
FBS: Fetal bovine serum
FCS: Fetal calf serum
GGT: Gamma-glutamyltranspeptidase
HFF-1: Human foreskin fibroblasts 1
HSCC: Hypopharyngeal squamous cell carcinoma
ICG: Indocyanine green
LED: Light-emitting diode
LMU: Ludwig Maximilian University of Munich
NA: Numerical aperture
NIR: Near-infrared
PBS: Phosphate buffered saline
SCC: Squamous cell carcinoma
SCC-UADT: Squamous cell carcinomas of the upper aerodigestive tract
SEM: Standard error of the mean
TBR: Tumor-to-background ratio
UKE: University Medical Center Hamburg-Eppendorf
VEGF-A: Vascular endothelial growth factor A
