# Supplementary material for: Targeting EpCAM expression via near-infrared fluorescent antibodies enables microscopic delineation of primary and recurrent HNSCC
Source: BMC Cancer. 2026 May 25;26:671. doi: 10.1186/s12885-026-16172-2 (PMC13200314; doi:10.1186/s12885-026-16172-2)
Supplement: Supplementary file 1 — Supplementary Material 1. [file 12885_2026_16172_MOESM1_ESM.docx]

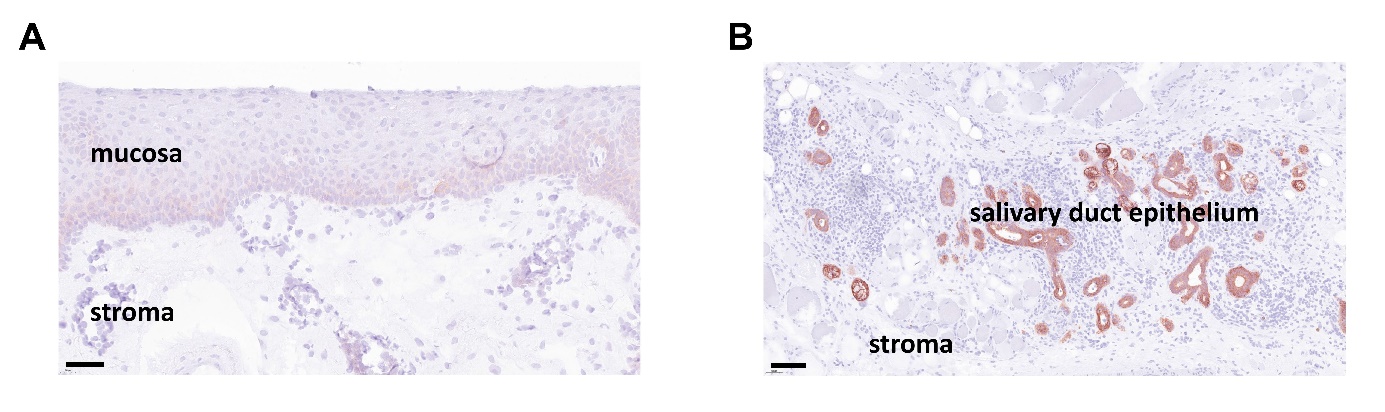


**Supplementary Figure 1. Representative images of EpCAM-positive tissue structures additional to HNSCC in patient tissue samples with primary and recurrent HNSCC. (A) Representative light microscopy image of marginally EpCAM-positive non-malignant mucosa (**scale bar: 60 µm) and **(B) representative light microscopy image of EpCAM-positive salivary gland tissue (**scale bar: 100 µm**) of immunohistochemically stained HNSCC tissue samples (**hematoxylin staining of nuclei; anti-EpCAM-antibody VU1D9: red)**.**


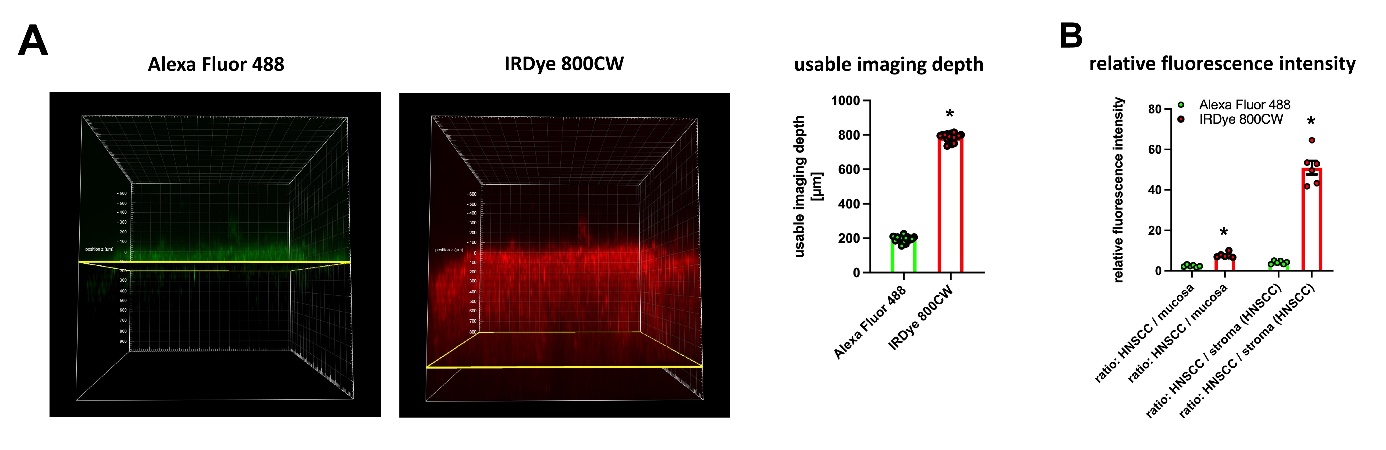


**Supplementary Figure 2. Comparison of the imaging properties of fluorescence immunostainings with either a dye emitting in the visible light range (AlexaFluor488) or a near-infrared dye emitting in the near-infrared light range (IRDye800CW). (A) Representative images and quantitative data on the usable imaging depth of immunostainings with the anti-EpCAM-antibody MT201 labeled either with AlexaFluor488 (visible light range) or IRDye800CW (near-infrared light range) as assessed by confocal laserscanning microscopy (scale bar: 50 µm; mean ± SEM for n = 15; * p < .05 vs Alexa Fluor 488). (B) Quantitative data on the relative fluorescence intensity ratios of HNSCC to non-malignant mucosa and of HNSCC to HNSCC-associated stroma as assessed in immunostainings with the anti-EpCAM-antibody MT201 labeled either with AlexaFluor488 (visible light range) or IRDye800CW (near-infrared light range) by confocal laserscanning microscopy (scale bar: 100 µm; mean ± SEM for n = 6; * p < .05 vs Alexa Fluor 488).**


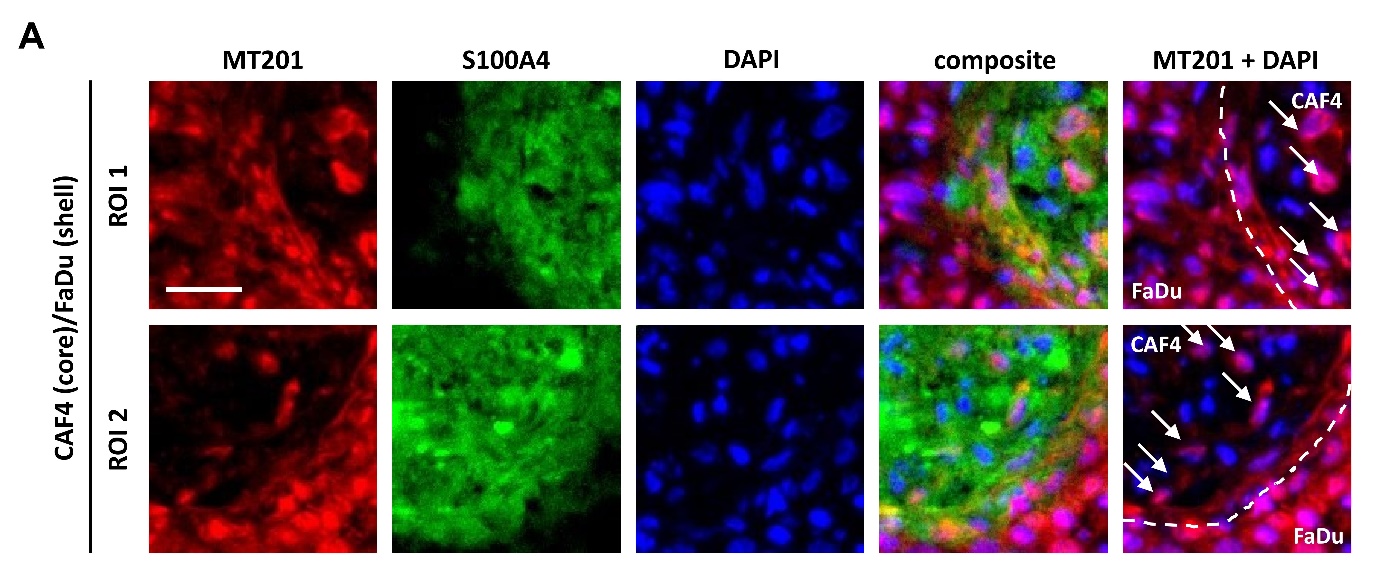


**Supplementary Figure 3. Representative images of single HNSCC cells infiltrating into the HNSCC-associated fibroblast area in bicellular core-shell-spheroids of HNSCC and HNSCC-associated fibroblast cells. (A) Two representative regions of interest extracted from a multi-color epifluorescence microscopy image of an immunohistochemically stained cryo-section of a bicellular core-shell-spheroid of HNSCC (FaDu; shell) and HNSCC-associated fibroblasts (CAF-4; core) illustrating single EpCAM-positive HNSCC cells (white arrows) infiltrating into the S100A4-positive HNSCC-associated fibroblast area (scale bar: 30µm; EpCAM/MT201: red; S100A4/fibroblasts: green; DAPI/DNA: blue; white dashed line: FaDu/CAF-4 border).**


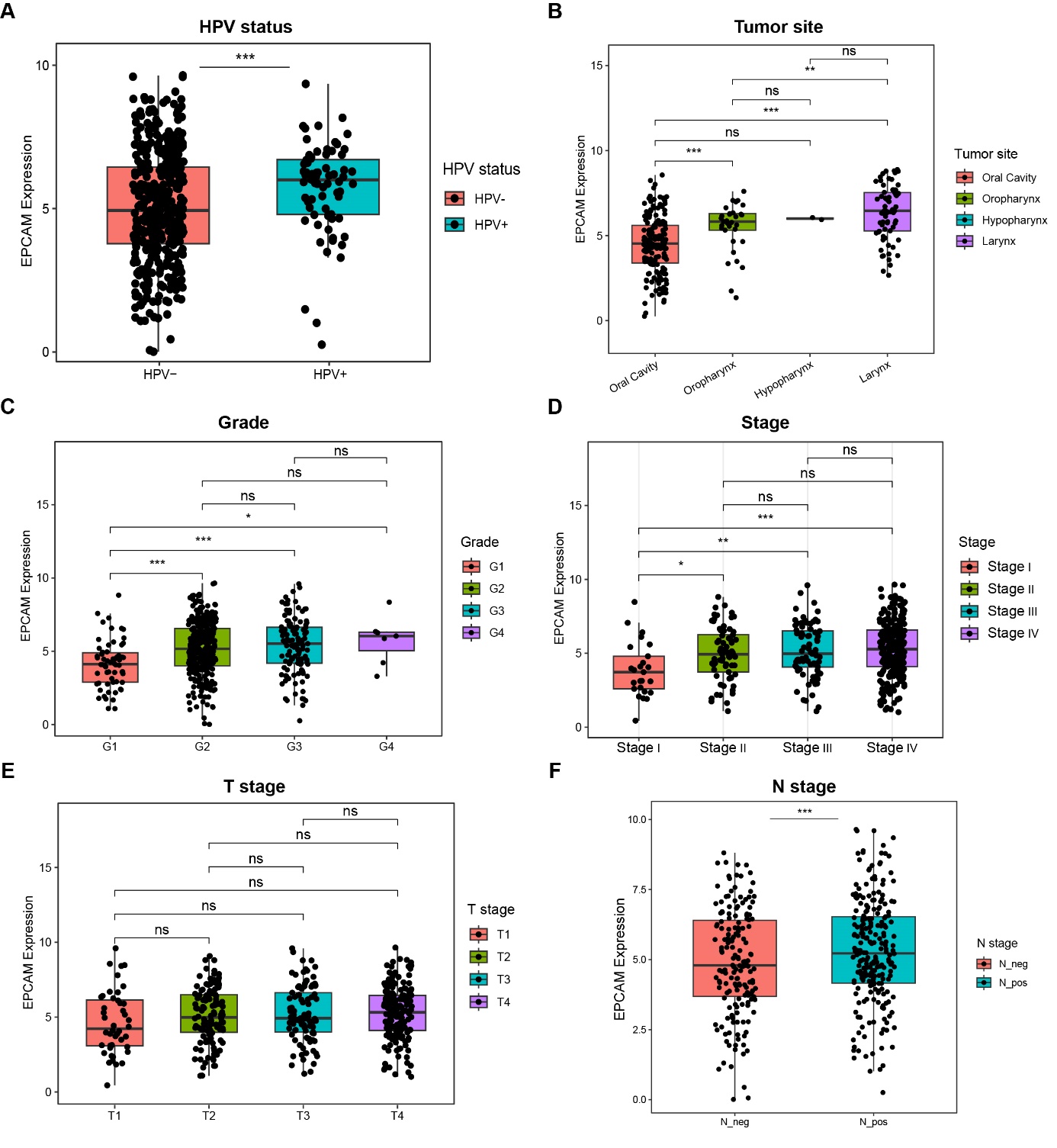


**Supplementary Figure 4. Association between EpCAM RNA levels and clinicopathological characteristics of HNSCC.** **(A)** EpCAM RNA levels in HPV-negative (HPV−) and HPV-positive (HPV+) tumors. **(B)** EpCAM RNA levels according to tumor site (oral cavity, oropharynx, hypopharynx, and larynx). **(C)** EpCAM RNA levels stratified by histological grade (G1–G4). **(D)** EpCAM RNA levels according to clinical stage (Stage I–IV). **(E)** EpCAM RNA levels according to T stage (T1–T4). **(F)** EpCAM RNA levels according to lymph node status (N− vs. N+). (Statistical differences were analyzed using the Wilcoxon rank-sum test. For multiple comparisons, p values were adjusted using the Benjamini-Hochberg (BH) method. ns, not significant; *p < 0.05; **p < 0.01; ***p < 0.001).

**Supplementary Table 1.** **Tumor meta-data of HNSCC pathology of patients analyzed within this study from the LMU cohort.** **Table with tumor meta-data for the HNSCC pathology of all patients analyzed in this study (localization; p16-status; grading; TNM classification; parameters for HNSCC invasiveness).**

| **Primary HNSCC** | | | | | | | |
| --- | --- | --- | --- | --- | --- | --- | --- |
| **#** | **localisation** | **p16-status** | **grading** | **TNM** | **V-status** | **L-status** | **Pn-status** |
| 1 | **oropharynx** | **p16^+^** | **N/A** | **pT2 pN1 cM0** | **V0** | **L0** | **Pn0** |
| **2** | **oropharynx** | **p16^+^** | **N/A** | **pT1 pN0 cM0** | **V0** | **L0** | **Pn0** |
| **3** | **oropharynx** | **p16^-^** | **G2** | **pT2a pN1 cM0** | **V0** | **L1** | **Pn0** |
| **4** | **oropharynx** | **p16^-^** | **G2** | **pT1 pN0 cM0** | **V0** | **L0** | **Pn0** |
| **5** | **oropharynx** | **p16^-^** | **G2** | **pT2 pN0 cM0** | **V0** | **L0** | **Pn0** |
| **6** | **larynx** | **N/A** | **G2** | **pT1 pN1 cM0** | **V0** | **L0** | **Pn0** |
| **7** | **larynx** | **N/A** | **G2** | **pT2 pN0 cM0** | **V0** | **L0** | **Pn0** |
| **8** | **oral cavity** | **N/A** | **G2** | **pT1 pN0 cM0** | **V0** | **L1** | **Pn0** |
| **9** | **hypopharynx** | **N/A** | **G3** | **pT4a pN2a cM0** | **V0** | **L1** | **Pn0** |
| **Recurrent HNSCC** | | | | | | | |
| **#** | **localisation** | **p16-status** | **grading** | **TNM** | **V-status** | **L-status** | **Pn-status** |
| **10** | **oropharynx** | **p16^-^** | **G3** | **rpT2 cN0 cM0** | **V0** | **L0** | **Pn1** |
| **11** | **oropharynx** | **p16^-^** | **G2** | **rpT4a pN0 cM0** | **V0** | **L1** | **Pn1** |
| **12** | **hypopharynx** | **N/A** | **G2** | **rpT4a cN0 cM0** | **V0** | **L0** | **Pn0** |
| **13** | **neopharynx (larynx)** | **N/A** | **G3** | **rpT1 pN0 cM0** | **V0** | **L1** | **Pn0** |
| **14** | **oropharynx** | **p16^-^** | **G3** | **rpT4a pN1 cM0** | **V0** | **L1** | **Pn0** |
| **15** | **oropharynx** | **p16^-^** | **G2** | **rpT3 cN0 cM0** | **V0** | **L1** | **Pn1** |
| **16** | **larynx** | **N/A** | **G3** | **rpT4a cN0 cM0** | **V0** | **L1** | **Pn1** |
| **17** | **larynx** | **N/A** | **G2** | **rpT3 cN0 cM0** | **V0** | **L0** | **Pn0** |
| **18** | **larynx** | **N/A** | **G3** | **rpT4a pN0 cM0** | **V0** | **L0** | **Pn1** |

**Supplementary Table 2.** **Tumor meta-data of HNSCC pathology of patients analyzed within this study from the UKE cohort.** **Table with tumor meta-data for the HNSCC pathology of all patients analyzed in this study (localization; p16-status; grading; TNM classification; parameters for HNSCC invasiveness).**

| **Primary HNSCC** | | | | | | | |
| --- | --- | --- | --- | --- | --- | --- | --- |
| **#** | **localisation** | **p16-status** | **grading** | **TNM** | **V-status** | **L-status** | **Pn-status** |
| 1 | **larynx** | **p16^-^** | **G2** | **pT2 pN1 cM0** | **V0** | **L1** | **Pn0** |
| **2** | **oropharynx** | **p16^-^** | **G2** | **pT2a pN1 cM0** | **V0** | **L0** | **Pn0** |
| **3** | **oropharynx** | **p16^-^** | **G2** | **pT1 pN0 cM0** | **V0** | **L0** | **Pn0** |
| **4** | **larynx** | **p16^-^** | **G2** | **pT2 pN0 cM0** | **V0** | **L1** | **Pn0** |
| **5** | **larynx** | **N/A** | **G2** | **pT1 pN1 cM0** | **V0** | **L0** | **Pn0** |
| **6** | **oropharynx** | **p16^+^** | **G2** | **pT2 pN0 cM0** | **V0** | **L0** | **Pn0** |
| **7** | **hypopharynx** | **p16^-^** | **G2** | **pT2a pN1 cM0** | **V0** | **L1** | **Pn0** |
| **8** | **larynx** | **p16^-^** | **G3** | **pT1 pN0 cM0** | **V0** | **L0** | **Pn0** |
| **Recurrent HNSCC** | | | | | | | |
| **#** | **localisation** | **p16-status** | **grading** | **TNM** | **V-status** | **L-status** | **Pn-status** |
| **9** | **oropharynx** | **p16^-^** | **G2** | **rpT2 cN0 cM0** | **V0** | **L1** | **Pn0** |
| **10** | **larynx** | **p16^-^** | **G3** | **rpT4a pN0 cM0** | **V0** | **L1** | **Pn1** |
| **11** | **larynx** | **N/A** | **G3** | **rpT3 cN0 cM0** | **V0** | **L0** | **Pn0** |
| **12** | **oropharynx** | **p16^+^** | **G3** | **rpT4a cN0 cM0** | **V0** | **L1** | **Pn1** |
| **13** | **larynx** | **p16^-^** | **G2** | **rpT3 pN0 cM0** | **V0** | **L0** | **Pn1** |
| **14** | **oropharynx** | **p16^-^** | **G3** | **rpT4a cN0 cM0** | **V0** | **L1** | **Pn0** |
